# Assessing the efficacy of tablet-based simulations for learning pseudo-surgical instrumentation

**DOI:** 10.1371/journal.pone.0245330

**Published:** 2021-01-14

**Authors:** James H. Kryklywy, Victoria A. Roach, Rebecca M. Todd

**Affiliations:** 1 Department of Psychology, University of British Columbia, Vancouver, British Columbia, Canada; 2 Department of Foundational Medical Studies, Oakland University William Beaumont School of Medicine, Rochester, Michigan, United States of America; 3 Department of Surgery, Oakland University William Beaumont School of Medicine, Rochester, Michigan, United States of America; 4 Dajvad Mowafaghian Centre for Brain Health, University of British Columbia, Vancouver, British Columbia, Canada; National University of Singapore, SINGAPORE

## Abstract

Nurses and surgeons must identify and handle specialized instruments with high temporal and spatial precision. It is crucial that they are trained effectively. Traditional training methods include supervised practices and text-based study, which may expose patients to undue risk during practice procedures and lack motor/haptic training respectively. Tablet-based simulations have been proposed to mediate some of these limitations. We implemented a learning task that simulates surgical instrumentation nomenclature encountered by novice perioperative nurses. Learning was assessed following training in three distinct conditions: tablet-based simulations, text-based study, and real-world practice. Immediately following a 30-minute training period, instrument identification was performed with comparable accuracy and response times following tablet-based versus text-based training, with both being inferior to real-world practice. Following a week without practice, response times were equivalent between real-world and tablet-based practice. While tablet-based training does not achieve equivalent results in instrument identification accuracy as real-world practice, more practice repetitions in simulated environments may help reduce performance decline. This project has established a technological framework to assess how we can implement simulated educational environments in a maximally beneficial manner.

## Introduction

Designing training protocols that effectively prepare people for rare or dangerous situations is not a trivial task. Skills and knowledge required by perioperative nurses cannot always be obtained through repetition-based practice, which can expose patients to unnecessary risk and is not feasible for less common procedures. Perioperative nursing education, like the majority of other nursing domains, often depends on text/image-based memorization of surgical instruments as a method of self-directed learning outside the operating room [1–4]. This approach is beneficial for patient care, yet it limits the synthesis of concrete information, and fails to train nurses in every aspect of the required tasks, as it lacks both the interactive and ordered components of a surgical procedure [5].

Recent advancement of digitally-simulated training environments (e.g., tablet-based programs and immersive virtual reality) provide one promising avenue to expand healthcare training. In addition to reducing the risk to patient populations, and increasing the opportunities for practice of rare procedures, digitally-simulated training environments allow greater accessibility to training than real-world practice, while maintaining some of the interactive components not present during text-based study. In particular, tablet-based training can afford trainees an opportunity to practice procedures easily at home, during commutes, or even as a refresher immediately before performing an actual task. Yet, while simulated environments have been implemented with great success in multiple training situations—semi-realistic multisensory simulations have been in use for nearly fifty years [see 6 for review]—and are demonstrated to be highly effective as educational tools in the medical profession [7–10], the generalizability of skills from tablet-based learning platforms remains unknown. They lack a history of implementation and evaluation—particularly those utilized in perioperative nursing education [see 11 for review]. For example, a recent meta-analysis of the viability of digital education tools [12] identified only *seven* empirical studies assessing the validity of tablet-based education, yet there is a growing market push for their implementation in the classroom [13–16]. This highlights a vital need to assess the utility of tablet-based training tools, particularly those, involving low-fidelity simulation (e.g., tablet-based procedure simulations), which are applied as educational tools for complex, high-risk procedures.

A critical consideration for the development and implementation of tablet-based simulations is that they do not demand the haptic interaction required of real-world tool use, and thus may not train the same neurocognitive mechanisms [17]. When interacting with tangible objects, humans employ visual search strategies that leverage the features of the three-dimensional (3D) world to identify tools; however, when the same tools are presented virtually on a tablet, the dimensionality is decreased to two (2D). This reduction has been demonstrated to both alter the manner in which we process visual information in real time [18], and influence our memory of, and attention towards, non-tangible objects [19, 20]. A contributing factor to these differences in performance could be neural systems recruited for target-directed actions towards tangible versus digital objects. For example, neuroimaging studies have demonstrated that pantomimed tool use, when performed adjacent to the actual tool, activates dissociable neural regions from those activated by the use of the tool itself [21]. The dissociability of neurocognitive recruitment during interactions with digital versus real-world objects suggests that these tasks may not be as interchangeable as they appear. The haptic feedback we receive when handling real-world items is integral to our neural representation of that object, regardless of the equivalency of visual precepts [22]. Thus, training skilled procedures through virtual simulations, particularly those with lower real-world fidelity, such as tablet-based simulation, may not actually lead to improvements in real-world task performance.

Given the increasing push for the integration of tablet-based simulation training into healthcare education, it is imperative that we assess its educational value relative to our existing training methods. In the current study, we directly compare real-world performance of an interactive procedure following pseudo-surgical instrumentation training (i.e., instrumentation training for items similar to, but not actually surgical instruments) through 1) tablet-based simulation, 2) text-based studying, or 3) real-world practice. In order to accurately assess the current methodologies available in the field, we modeled training protocols in both self-guided training environments on educational tools that are currently commercially available and marketed for the training of perioperative nurses [4, 15]. Thus, we compared the learning process across both interactive training procedures to identify relative strengths and weaknesses in the application of each. We predicted that test performance following tablet-based training and real-world practice would elicit significantly greater accuracy and shorter response times relative to text-based study. This prediction is consistent with evidence of enhanced recruitment of neural systems involved in motor responses for non-textual learning, as well as the potential influences of additional procedural ordering information present during tablet-based training and real-world practice compared to text-based study. Additionally, we predicted an increase in task repetitions during tablet-based training compared to real-world practice, consistent with the minimization of time required for instrument and room set-up.

## Methods

### Participants

Forty-six subjects were recruited to participate in the experiment from undergraduate courses in the Department of Psychology at the University of British Columbia. All participant gave written informed consent prior to beginning the experiment. Notably, this population displays a high degree of similarity across sex, ethnicity, and age with students in an undergraduate healthcare provider degree program [23, 24]. Due to technical error during test sessions (e.g., no video recorded data available to be analysed) or attrition, data from ten subjects were not available for assessment of test/retest performance, leaving thirty-six subjects (26 female; 33 right-handed) with a mean age of 20.1 (range 18–32, SD 2.44) for our primary test-retest analyses. For analyses of learning dynamics, data from two subjects were removed from the initial sample, due to technical error during training, leaving forty-four subjects, (29 female, 37 right-handed) with a mean age of 19.9 (range 18–32, SD 2.37). Target sample size was determined by power analysis in the statistical software G*power [25]. Due to the limited prior pertinent literature available to estimate effect size, a conservative effect size estimate of (.2) and power of 0.8 were used for this calculation. This resulted in a target sample of 42 participants for our primary analysis: a 3 (training-type: text, tablet, real-world) X 2 (time: immediate vs delayed test) repeated measures (rm) ANOVA). All participants had normal hearing, normal or corrected-to-normal vision, were fluent English speakers (>85% indicating English as a first language) and had completed an average of 13.5 years of formal education (range 12–18, SD 2.13). The study was approved by the Behavioural Research Ethics Board at the University of British Columbia.

### Stimuli and apparatus

In an effort to emulate the types of instruments encountered by perioperative nurses in the operating room, 105 unique handheld items were chosen as “pseudo-surgical” instruments. Pseudo-surgical instruments were chosen to ensure the safety of all researchers and participants, and ranged in size from 30–219 mm along the longest axis. To ensure that no prior knowledge of the item could influence results, each instrument was given a novel name, modelled after the structure of those in Wells’ *Surgical Instruments*: *A pocket guide* [4]. As is typical of conventional surgical instruments, pseudo-surgical instruments were named to include information on their structure, function, or [fictional] developer of the item. Within this naming system, there were sub-groups of functional or linguistic classes (e.g, pressers, imbudos etc., see S1 Table for full item names). Pseudo-surgical instruments were divided into three separate sets of 35 to allow for a unique set to be used in each of the three unique training environments (see below). While participants were exposed to all 35 pseudo-surgical instruments during training, only twenty-one items from each set were called during each procedure, and this was consistent across training protocols. Sub-family names were kept consistent within, but not between, sets to minimize transfer of learning between training environments.

**Fig 1:**
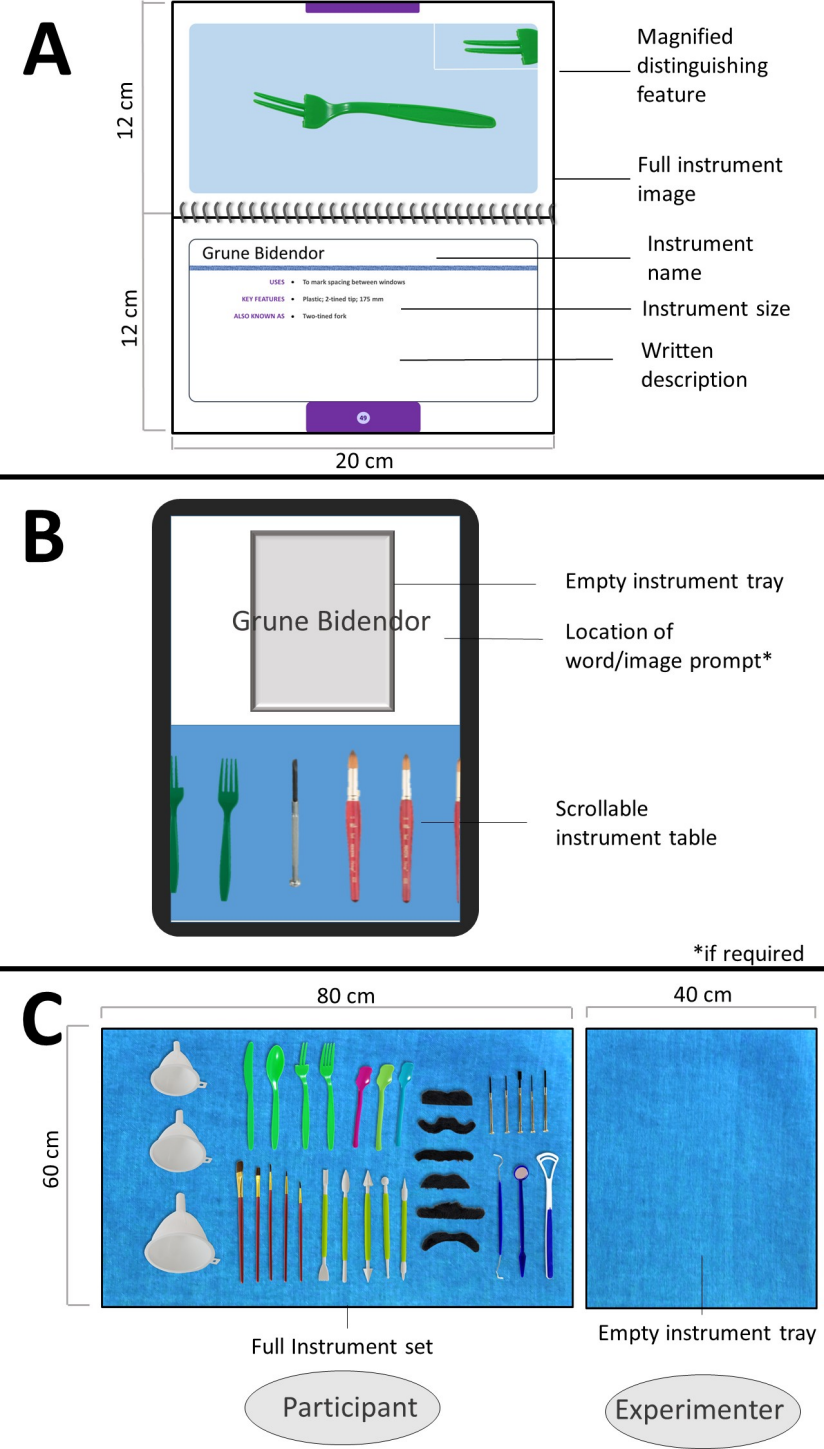
Training protocols. Participants trained with three separate training structures prior to performing real-world assessment of acquired knowledge. A) Text-based study tools consisted of a flash card for each item that included two images (full instrument and an enlargement of the distinguishing feature), and a text-based description including item uses, physical characteristics, and alternate names. B) The tablet-simulated environment consisted of items of a given set of instruments presented on the lower half of the tablet screen (4 viewable at once, with the remaining visible by scrolling left/right), and an empty ‘surgeon’s’ tray presented above. Following an auditory request, participants were required to swipe the instrument into the empty tray. Text, then image hints were provided if the correct idem was not identified within a given period. C) For real-world procedures (real-world training + *ALL* test procedures), pseudo-surgical instruments were arranged in a predefined order on a ‘surgical prep table’ located directly in front of the participant. Following correct identification, instruments were placed by the experimenter on an empty workspace. Incorrect instruments were returned to the prep table.

To assist the training condition in a manner analogous to current text-based learning protocols, a study guide of text and photo based flash cards was developed for each of the three pseudo-surgical instrument sets (Fig 1A) modeled on Wells’ *Surgical Instruments*: *A pocket guide* [4]. This study-guide included two photographs of each item, one of the full item and one close up of the critical feature, as well as a written description of the object’s size and function. Of note, while there was also information on to the physical description of items in the tablet-based simulations and real-world items, this information was not made explicit through text descriptions but was provided in the form of interactive explorable objects. All tablet-based tasks were performed on a 9.7’ iPad Air 2 (Model 1566; Apple Inc.). Simulated training environments were modified from the Conquer Experience^TM^ surgical instrumentation platform for tablets, PeriopSIM [15].

### Training procedures

To optimize power and eliminate confounds due to individual differences between participants we used a within-subject design. Participants were trained to recognize each of the three pseudo-surgical instrumentation sets in a distinct training environment: text-based study, tablet-based game-play, and real-world item/surgeon interaction, described below. To ensure that no order or practice effects could emerge in the group data, the assignment of instrumentation-set to training environment was randomized between participants, as was the order in which each participant would experience each learning environment. Participants were afforded 30 minutes in each training environment to learn the names, and where applicable, the procedural order of the pseudo-surgical instruments.

#### Text-based study

In the text-based condition, participants were given a set of flash cards corresponding to one of the pseudo-surgical instrumentation sets to assist instrument identity learning. They were instructed to read through and memorize all the names of all pseudo-surgical instruments in the time provided, with the explicit understanding that they would be tested on their ability to identify the items following training. To reflect realistic study environments, no additional instructions were given until after the training period; the participant was entirely responsible for sustained attention to the study material in this training environment. Furthermore, no information about the order in which the tools would be requested (i.e., procedural order) or handling and manipulation of the items, virtual or otherwise, was provided during text-based study, consistent with information given in currently available instrumentation study guides [4].

#### Tablet-based simulation

The tablet-based simulation training environments employed in this study were modeled after a commercially available application used to train perioperative nurses on surgical instrumentation [15]. After being familiarized with the tablet-based application (Fig 1B), participants were instructed to work through the instrumentation procedure as many times as possible in the time provided. Participants had to identify 21 instruments from a display of 35 in a pre-determined order by dragging the requested instrument from the lower instrumentation tray to the empty tray above. Each trial began with an auditory verbal request for an instrument. If no response, or an incorrect response, was made within 3000 ms, the name of the requested item would appear in text over the empty tray. If no response was made after an additional 3000 ms, the text prompt was replaced with an image of the instrument. This remained visible until the correct instrument was swiped to the empty tray. Following the identification of the requested item, participants would receive an auditory prompt indicating whether the response was correct (e.g., “That is correct” or “No, not that one”). Within the application, participants were awarded points based on their speed and accuracy in correctly moving the requested instrument to the empty tray. After each play-through of 21 instruments, there was a 2500 ms pause before re-setting to the beginning of the simulated procedure. Importantly, in this protocol, participants were still required to make target-directed actions towards requested instruments in the same procedural order as would be demanded by a real-world task. Participants were instructed to work through the simulated training environment as many times as possible in the allocated training time.

#### Real-world practice

In the real-world condition, instrumentation training using real-world interaction was conducted to directly emulate the task demands of a perioperative nurse in the OR. Following a specific predefined procedural order, the experimenter requested 21 of the 35 presented instruments, each of which were to be placed in the experimenter’s open hand. After placing the instrument into the palm of the experimenter, the participant received verbal feedback on whether the instrument was correct. Correct instruments would be taken by the experimenter and placed on an empty workspace while incorrect instruments were taken back by the participant and placed back on the ‘prep table’ (Fig 1C). For the initial two training runs, where participants were necessarily naïve to the naming system of the instrumentation set, the experimenter could give a verbal hint about the instrument being requested if the participant was unable to identify the instrument after ~10 s (e.g., wrong class) in a manner consistent with the automated prompts of the tablet-based protocol. For the remaining repetitions, experimenter feedback was limited to indications of item correctness. Following each run through all 21 instruments, the experimenter would reset the ‘surgical prep table’ to its initial layout (~30 s) and begin the procedure again. Participants were requested to complete as many iterations of the procedure as possible in the training period. All real-world training sessions were video recorded for further data processing.

### Testing procedure

To assess the efficacy of each training environment for real-world procedural learning, after each half-hour training period, participants completed an instrument identification task with tangible pseudo-surgical instruments emulating objects and protocols experienced in real-world operating rooms. Here, participants were presented with instruments identical to those on which they had been trained (following the same procedure as in the real-world training) and were required to pass individual instruments to the experimenter upon request as quickly and as accurately as possible. If the response was incorrect, participants were required to continue passing instruments until the correct object was identified. Experimenter feedback during the test phase was limited to a verbal indication of accuracy (i.e., correct/incorrect).

Finally, to assess long-term retention of instrument names and procedural ordering for each trained instrumentation set, participants were invited back into the laboratory one week after initial training. While the order of assessment was identical to the training order from week one, participants were not given any time for re-training to familiarize themselves with the instruments prior to this testing session. All test sessions were video recorded for further data processing. Following all experimental tasks (i.e., after the delayed retention test), participants completed a subjective evaluation of the training protocols, rating each method for its perceived *effectiveness*. Additional ratings of the tablet- and text-based training methods were collected assessing the participant’s *motivation to use* each training task, as well as their belief in the likelihood that they would *continue to use* each method in a classroom setting. All ratings (*Perceived Effectiveness*, *Motivation to Use*, and *Likelihood of Continued Use*) were collected on a visual analog scale ranging from “*not at all”* to “*very”* that was divided into 100 points for subsequent analysis.

### Analyses

The behavioural measures of interest from both week one and week two testing sessions were accuracy of instrument identification (i.e., % correctly identified on the first attempt), average number of errors (incorrect trials), and response time to instrument hand-off (correct trials only, measured from the end of the verbal request to the moment the item touches the experimenter’s palm). All dependent measures from the real-world training and test sessions were extracted from the video recordings. Each video session was viewed by two experimenters (video raters) with explicit knowledge of all instruments, protocols and request orders required. Importantly, while raters had knowledge of the experimental design, and the items contained in each set, they were blind to the specific instrument set-training protocol pairing during data extraction for all test and retest video sessions. All video raters were trained to identify the onset of a trial (i.e., end of the verbal prompt), the end of a trial (i.e., when the instrument contacts the experimenter hand), and the procedural order and naming system for all instruments to assess accuracy and errors. A custom semi-automated video segmentation program was used to assist video data extraction which allowed for manual tagging of trial onset and offset, as well as trial outcome (i.e., correct or incorrect) for automated calculation of response time and for session averaging. As an interclass correlation analysis performed on the test procedure video sessions (data from immediate and delayed retention testing accuracy for all training conditions) found excellent internal consistency between video raters (Cronbach’s alpha = 0.950), data from Rater 1 was used for all subsequent analyses.

As each participant completed training (and subsequent testing) in all three training protocols in a fully within-subject design, a 3 (training-type: text, tablet, real-world) X 2 (time: immediate vs delayed retention test) repeated-measures (rm) ANOVA was conducted on each of the outcome measures using IBM-SPSS [26]. Main effects and interactions were further investigated using the estimated marginal means (MM). All *p*_*MM*_ values presented have been corrected for multiple comparisons using a Bonferroni correction. Additionally, a Greenhouse-Geisser correction has been performed to address any instances where an assumption of sphericity for these data could not be made. Non-significant effects were further investigated through a series of two one-sample t-tests (TOSTs) for statistical equivalence [27–29] using R [30]. All *p*_*TOST*_ values presented have been corrected to control for family-wise error rates [31], with *p*_*TOST*_ < 0.05 indicative of statistical equivalency between conditions [28].

To investigate more dynamic learning processes in both the real-world and tablet-based training environments, and gain additional insight into the strengths and weaknesses of each, task performance during training sessions was analysed using non-parametric local regression (LOESS). In order to assess the course of learning over time in each training protocol, we examined a measure of peak performance achieved in each learning environment as well as the latency to that peak by both task repetition and time spent in the learning environment. Instrument identification accuracy data acquired during the learning periods were divided into ten distinct bins for both procedural repetition (1 bin = 1 task repetition), and training time (1 bin = 3 minutes), resulting in four distinct conditions (Tablet-simulation: repetition and timing, and Real-world: repetition and timing). To determine the accuracy and latency of peak performance in each condition, LOESS curves [32] were fitted to our accuracy data as a function of repetition/training time using a symmetric smoothing parameter of 0.5. Following this, we determined the bin in which maximum accuracy was obtained in the fitted data, and defined our peak performance threshold as one standard deviation below this value. To compare between conditions, we constructed a 95% bootstrapped bias-corrected and accelerated confidence interval around this point estimate. All LOESS curve fitting and bootstrapping procedures were conducted in R [30] and modeled after those described in Meier and Giaschi [32].

## Results

### Subjective ratings

Following completion of all experimental procedures (i.e., post delayed retention testing), participants rated all three training environments for their perceived effectiveness. A 1 X 3 (training-type: text, tablet, real-world) repeated-measures ANOVA performed on these data identified a significant main effect of training (F_(2,70)_ = 6.224, *p =* 0.003). Real-world training ($\bar{x}$ = 80.50, sd = 15.58) was perceived as more effective compared to either text- ($\bar{x}$ = 63.19, sd = 23.48) or tablet- ($\bar{x}$ = 69.33, sd = 19.93) based training (*p*_MM_ = 0.010 and *p*_MM_ = 0.032 respectively). No differences were identified between tablet- and text-based training (*p*_MM_ = 0.732). An equivalence test marginally supported this null result, indicating trend-level equivalence for these conditions (*p*_TOST_ = 0.058).

Ratings of the tablet- and text-based training methods assessing participants’ motivation to study in each training task, and their likelihood of implementing each training tool in a classroom setting, revealed no differences between these training conditions (all p_MM_s > 0.277) and marginal support for equivalence (all *p*_TOST_s < 0.062).

### Test performance

Performance on real-world procedures was assessed by three separate criteria: accuracy of instrument identification (% correct), response time (correct trials only), and number of errors (incorrect trials only) (Table 1). Each measure was subject to a 3 (training-type: text, tablet, real-world) X 2 (time: immediate vs delayed test) repeated measures ANOVA.

**Table 1:** Performance across training conditions and assessment timing.

Training Protocol	Test Session	Accuracy^a^			Response time^b^			Errors^c^		
		Mean	St.Dev.	Range	Mean	St.Dev.	Range	Mean	St.Dev.	Range
Text	*immediate*	.836	.103	.50–1.00	2.59	0.933	0.83–5.06	1.32	0.61	0.00–3.43
	*delayed*	.643	.159	.25-.91	4.42	3.54	2.06–23.61	2.29	1.42	1.00–7.25
Tablet	*immediate*	.845	.103	.5–1.00	2.59	0.93	0.83–5.06	1.31	0.61	0–3.43
	*delayed*	.652	.156	.36-.91	3.34	1.71	1.72–11.89	2.04	0.71	0.83–3.92
Real-world	*immediate*	.901	.182	.25–1.00	1.91	0.725	.68–3.44	0.836	1.05	0.00–4.33
	*delayed*	2.27	1.50	.27-.95	4.14	1.64	2.42–10.12	2.16	1.70	1.00–7.85

^a^*Accuracy* is presented as the proportion of items identified correctly on the first response.

^b^*Response time* is presented for correct trials only.

^c^*Errors* are measured as the number of incorrect responses prior to a correct response and is measured for incorrect trials only. Participants with a perfect test session were given an average error of 0.

**Fig 2:**
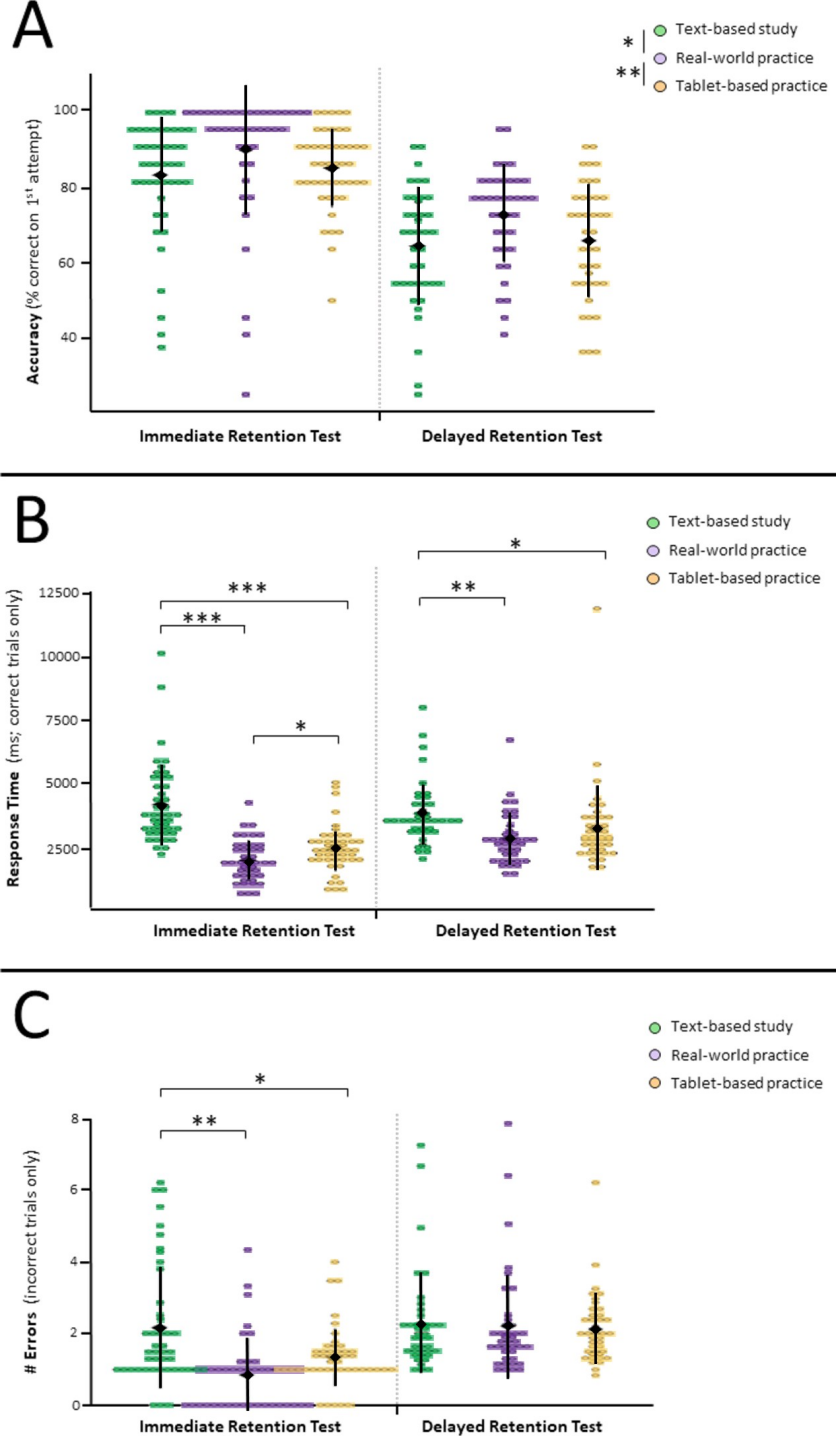
Performance of real-world test procedures immediately and 1-weeek delayed from training. For all plots, each dot represents an individual participant. A black diamond represents the population mean, with error bars indicating standard deviation. A) *Accuracy by training protocol*. Real-world-practice resulted in better instrument identification accuracy during retention testing than either tablet-based simulation training or text-based study. B) *Response time by training protocol*. Less time was required to perform the instrument hand-off following real-world-practice and tablet-based simulation compared to text-based study. C) *Errors by training protocol*. Differences in the number or errors per incorrect instrument were observed only during the immediate retention test. During delayed retention testing, all protocols resulted in equivalent errors. For all plots, individual subject data are shown by coloured circles, with the group mean and st. dev indicated in black.* *p* < 0.05, ** *p* < 0.01, *** *p* < 0.001, ^m^ *p* < 0.10.

For accuracy, main effects of both training-type (F_(2,70)_ = 6.375, *p =* 0.003) and time (F_(1,35)_ = 122.699, *p <* 0.001) were observed (Fig 2A). Instruments were identified with greater accuracy following real-world training compared to either tablet (*p*_MM_ = 0.049) or text-based (*p*_MM_ = 0.006) training. No differences in accuracy were noted between tablet and text-based training (*p*_MM_ = 1.00), a null finding supported by follow-up equivalency testing (*p*_TOST_ = 0.018). In addition, a general decrease in performance was observed from immediate to delayed test sessions (*p*_MM_ < 0.001). No training-type X time interaction was identified in the accuracy data (F_(2,70)_ = 0.043, *p* = 0.958) with follow-up equivalency testing confirming equivalency between tablet- and text-based training at both immediate and delayed testing (all *p*_TOST_s < 0.015), but not for all other contrasts (all *p*_TOST_s > 0.0248). This pattern of findings was analogous to that observed for the main effect of training type.

For response time, there was a main effect of training-type (F_(1.54,53.87)_ = 36.752, *p <* 0.001). Additionally, a trend-level effect of time emerged (F_(1,35)_ = 4.110, *p =* 0.05). Shorter response times during testing were observed following real-world and tablet-based training compared to text-based study (*p*_MM_ < 0.001 and *p* = 0.045 respectively). Shorter response times were also observed following real-world training compared to tablet-based training (*p*_MM_ < 0.001). Notably, a training-type X time interaction was identified (F_(1.74,61.02)_ = 4.574, *p* = 0.014; Fig 2B). This interaction was driven by differences between real-world and tablet-based training during immediate testing (*p*_MM_ = 0.011) but not during delayed testing (*p*_MM_ = 1.00; *p*_TOST_ = 0.008). Both interactive training protocols (tablet and real-world) produced shorter response times compared to text-based study during both immediate and delayed test sessions (all *p*_MM_s < 0.014).

Analysis of error rates identified significant main effects of both training-type (F_(2,70)_ = 5.072, *p =* 0.009) and time (F_(1,35)_ = 41.111, *p <* 0.001). Fewer errors were observed following real-world compared to text-based study (*p*_MM_ = 0.034) while marginally fewer errors were made following tablet-based training compared to text-based study (*p*_MM_ = 0.077), with no support of equivalency observed in TOST analyses for this contrast (*p*_TOST_ = 0.504). No differences were observed between real-world and tablet-based training (*p >* 0.10, *p*_TOST_ = 1.00). As with accuracy, a decline in overall test performance was observed from immediate to delayed testing (*p* < 0.001). A significant training-type X time interaction (F_(1.45, 50.92)_ = 6.712, *p =* 0.006; Fig 2C) was also observed. This was characterized by differences between all training types during the immediate test: Both real-world practice and tablet-based simulation resulted in lower error rates than text-based learning (*p*_MM_ = 0.001 and *p*_MM_ = 0.039 respectively. There was also trend-level indication of marginally fewer errors for real-world practice compared to tablet simulations upon immediate testing *(p*_MM_ = 0.05) consistent with no evidence of equivalency (*p*_TOST_ = 0.632). No significant differences were identified during the delayed retention test (all *p*_MM_s > 0.935). This null finding for the delayed retention testing was substantiated by equivalency tests demonstrating equivalency between the text-based and real-world training conditions (*p*_TOST_ = 0.006), and trend-level support for equivalency of tablet-based training with both traditional training methods (*p*_TOST_ = 0.056 for both comparisons).

### Learning dynamics

To assess the impact of procedural repetition on learning to identify instruments, instrument identification accuracy from both real-world and tablet-based training conditions were compared against target performance levels (100% accuracy). A series of analyses fit non-parametric local regression (LOESS) curves to the accuracy data as a function repetition/training time. By conducting this analysis in data divided by both the number of procedural repetitions and the amount of time spent undergoing training, the relative strengths and limitations of each protocol can be assessed. From these analyses, the point at which maximum accuracy was obtained in the fitted data, indicative of the transition from a knowledge acquisition task to skilled practice of the task. Together, these analyses contextualize observed differences in test session performance between conditions in the amount of time spent in skilled practice, as well as highlight any potential ceiling effects for each training technique.

### Accuracy by procedural repetition

**Fig 3:**
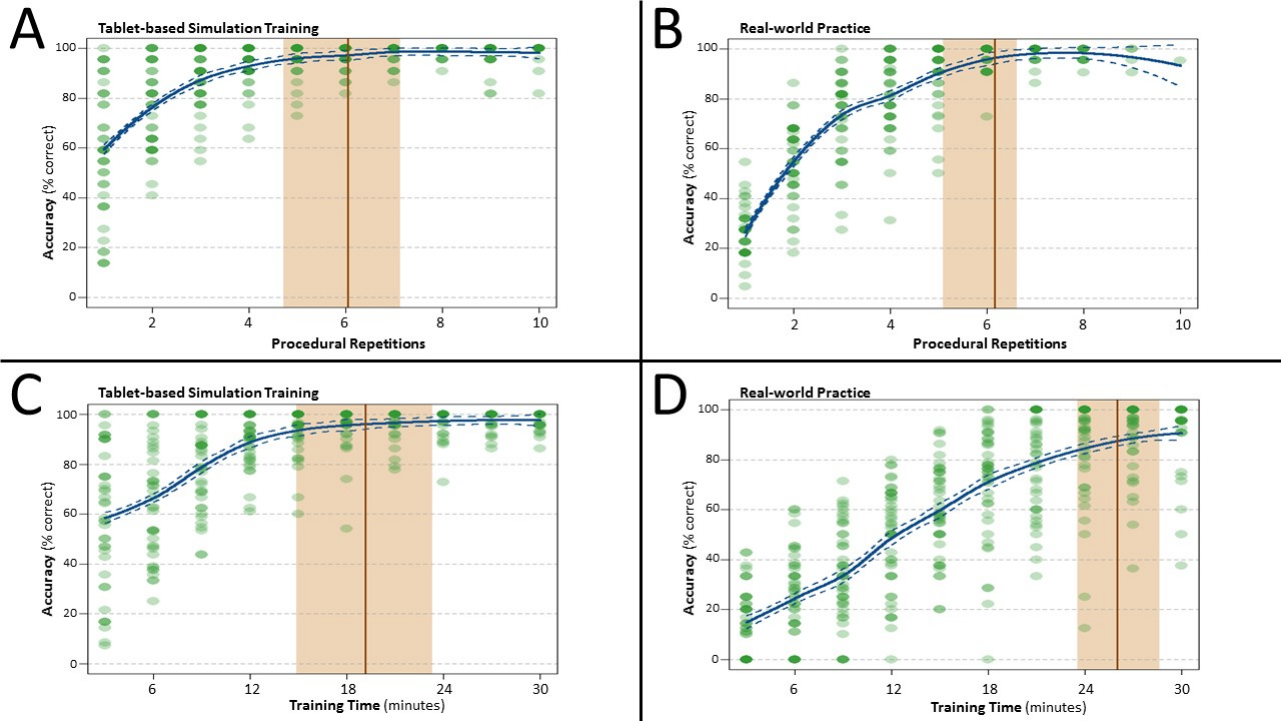
Instrument identification accuracy as a function of procedural repetition (A/B) and training time (C/D). Solid blue lines indicate the model LOESS fit (span = 0.5). The solid line represents the point estimate at which the LOESS model indicates that performance to be plateaued. The shaded area around this line indicates the 95% confidence interval (CI) around this estimate. Note that while highly overlapping point estimate CIs are observed between real-world and tablet-based training as a function of repetition, these CIs do not overlap as a function of training time, indicating that a performance plateau is reached significantly faster during tablet-based training.

In order to examine how many repetitions it took to reach peak participant accuracy across *task repetitions*, LOESS curves were constructed for data from both the tablet vs. the real-world training conditions. During tablet-based training, a performance plateau was observed after 6.06 procedural repetitions (Accuracy = 97.0%; *CI 95%* = 4.72–7.08 repetitions; Fig 3A). Notably, this was nearly identical in both number of repetitions and accuracy level to the learning pattern observed during real-world practice, where a performance plateau was observed after 6.16 procedural repetitions (Accuracy = 96.3%; *CI 95%* = 5.20–6.69 repetitions; Fig 3B). Yet while the number of repetitions required for peak performance did not differ between the tablet and real-world training environments, during the whole 30-minute training period there were more repetitions of the procedure for tablet-based simulation training compared to real-world practice (t_(39)_ = 6.017, *p* > 0.001).

### Accuracy by training time

To assess how long it took to reach peak performance as a function of *time*, LOESS curves were constructed for data from the training period segregated into ten 3-minute bins. During tablet-based training, peak performance was obtained at 19.2 minutes (Accuracy = 97.2%; *CI 95%* = 15.03–23.63 minutes; Fig 3C). In contrast, a later performance plateau was observed during real-world practice, with a performance plateau observed after 26.03 minutes (Accuracy = 87.4%; *CI 95%* = 23.73–28.62 minutes; Fig 3D).

## Discussion

There has been a recent push to incorporate mobile digital technologies into the occupational training of allied healthcare professions. One such application is the inclusion of tablet-based simulations as a replacement for, or augmentation of, traditional hands-on training for high-risk surgical procedures. The current study assessed real-world pseudo-surgical instrument identification following training with two established methods (text-based study and hands-on practice), and one emerging method (digital simulation). Performance using each of these methods was compared to performance following real-world practice. Notably, participants were significantly more accurate when identifying the instruments following real-world practice compared to both tablet-based simulation and text-based study. Immediate retention tests demonstrated a response time advantage for real-world training compared to simulation training, suggesting a more efficient nurse-surgeon interaction could result. Interestingly, however, this effect disappeared at test points one-week later, suggesting that tablet-based training may be less susceptible to performance decline than real-world training. To investigate the process of learning in each environment, additional analyses identified the level and latency of peak performance for each interactive training method. While the *number of repetitions* to reach peak performance was equivalent for tablet-based simulation and real-world training, net *time spent training* before reaching peak performance indicated that learners reached peak performance faster when practicing with tablet-based simulations. This finding suggests that with tablet-based training compared to real-world practice, less time is required to obtain knowledge for the procedures, thus more of the training period is spent practicing learned procedures.

### Learning instrument identity across training protocols

A substantial gap exists between marketing claims and scientific assessment of tablet-based training for healthcare professionals [33]. In the current study, real-world instrument identification was *less accurate* following tablet-based simulation than following real-world practice. However, in healthcare education, real-world practice is often not a viable method of training. Many procedures are either too resource intensive to be conducted solely as practice [e.g., 34, 35] or too complex or rare to allow trainees to learn in situ [36–38] given the potential risks involved [39]. Thus, early training of procedures is often conducted through observational [40, 41] or text-based learning [2–4]. In the current study, instrument identification accuracy following tablet-based training was equivalent to text-based training, yet both resulted in significantly lower performance compared to real-life training. This result is consistent with a body of literature suggesting a benefit of guided object-directed attention and actions for subsequent identification and recall of the object [for review, see 42]. It is also consistent with evidence of dissociable neural pathways for processing of both 2D versus 3D objects [18, 19] and object function versus object manipulation knowledge [43]. Overall, this work suggests that either tablet-based simulation or text-based study may be implemented as tool to augment, but not replace, real-world practice.

A potential avenue to overcome the performance deficit observed for 2D training approaches is the inclusion of interactive 3D visualizations for object and procedural learning [44–46]. The effectiveness of this manipulation, however, may depend on individual differences in spatial ability [47–50]. Furthermore, the addition of manipulatable 3D visualizations of instruments in tablet-based simulations may negate one of the advantages to tablet-based simulations suggested by the current study. Specifically, additional exploration time required to assess 3D instruments may eliminate the increased procedural repetitions observed for tablet-based training compared to real-world practice in the current work. Thus, the inclusion of interactive 3D visualizations may not produce the desired benefit in all learning domains [51]. Furthermore, while increasing the interactive model from 2D to 3D can enhance the fidelity of *visual* experience, it does not alter the *haptic* experience of the instrument; an additional benefit of real-world item handling well evidenced to enhance learning [22, 52]. Additional work is required to determine the level of visual dimensionality for objects presented in tablet-based training environments to ensure optimal accuracy in subsequent recall.

### Procedural efficiency across training protocols

Beyond accuracy, an additional marker of perioperative training effectiveness is the efficiency of nurse-surgeon interactions [53]. The current study operationalized this metric through both the response time for instrument hand-off (i.e., time from request completion, to the placement of the item in the ‘surgeon’s’ hand) and the number of response errors on incorrect trials. Whereas training in tablet-based simulations did not meet performance standards of real-world training for identification accuracy, interactive features of the task, including the number of errors and response times, were remarkably similar between the two, with both outperforming text-based study. The existence of sequential ordering information that is not present during text-based study may be one potential explanation for this performance discrepancy [54, 55]. Additionally, the object-directed behaviours required by both real-world and tablet-based training recruits and trains additional neural resources related to action-based processing which may act further instantiate learning of the procedures [21, 56, 57].

While current results indicate instrument interaction efficiency following real-world practice and tablet-based training was similar overall, one notable difference with respect to interaction efficiency emerged. During procedures tested immediately following the training session, response times were faster for real-world practice than tablet training (with both outperforming text-based study); however, when tested following a one-week latency period the observed difference between real-world and tablet-based training disappeared, and these conditions were statistically equivalent. The reduction in training advantage for real-world practice from immediate to delayed retention may stem from the increase in the number of repetitions performed during the tablet-based simulations following the attainment of a performance plateau. Practice repetitions following knowledge acquisition is well evidenced to strengthen learning of both semantic information [58, 59] and motor tasks [60–62], as well as simultaneous, but separate, cognitive and motor procedures [63]. Accordingly, the development of many digital learning platforms have relied nearly entirely on manipulating spaced-timing repetitions of the presented information [64]. For many of these platforms, considerable research has been conducted to investigate the temporal spacing structure required to maximize the learning benefit [65, 66]; this phenomenon has yet to be defined in respect to perioperative nursing procedures.

### Strengths and weaknesses of training methods

A notable difference between simulation training and real-world practice compared with text-based study, which may contribute to differences in interaction efficiency observed in the current work, is the specificity of items called and the ordering of items presented. Both tablet-based learning and real-world practice act as guided training for a single procedure, with presented instruments catered to those required for each specific task. In contrast, text-based study guides often include additional items beyond the required scope of a single surgical procedure. As a consequence, while training through tablet-based simulations may lead to improved performance on a *single task* compared to text-based study, the latter method may show enhanced *generalizability* of learned performance, though additional work is needed to assess this claim.

The two interactive training protocols further differ from text-based study in the extent to which multisensory information is presented during training. Beyond the haptic and motor feedback discussed above, in the tablet and real-world conditions, instrument requests during training are made through auditory prompts. To contrast, text-based study of the items required no external or motor feedback, with all novel information regarding the instruments limited to the words and images in the study guides provided. As noted above with respect to haptic information, the inclusion of this additional modality in the real-world training condition may reinforce instrument-specific knowledge [67, 68] by creating a more enriched cognitive representation of the item [22, 68, 69]. In particular, as the testing procedure requires the participant to respond to an auditory prompt to begin each trial, this information may become even more valuable for task performance, with the additional practice of parsing and processing the verbal request practiced as well. Together, these results highlight the importance of multiple sources of information to knowledge retention and may help explain the performance differences observed in the three training protocols of the current experiment. Following this, one avenue of interest for subsequent work involves the non-clinically relevant outcomes of the training procedures. In particular, results from the current work suggest that multisensory presentations of items led to enhanced identification of the items during subsequent test procedures. Notably, however, the final testing protocols were conducted in multisensory environments. Further work, shifting away from the real-world application of medical training, should investigate whether this increased performance would remain consistent in sensory minimized testing protocols, such as picture identification, or non-tactile gesturing.

Interestingly, current results may also suggest a potential ceiling for tablet-based simulation training protocols (in their current form) that is fundamentally lower than that of real-world practice for the clinically relevant skills. Even with the additional training repetitions performed in tablet simulations, identification accuracy lagged behind that of real-world practiced items in the current work. This effect may be mediated, in part, by the level of fidelity observed in the simulated environment [70–72]. Additional work investigating both repetition spacing and simulation fidelity is required to fully optimize healthcare simulation protocols.

## Conclusions

Tablet-based simulated training media are being increasingly incorporated into the education curriculum for healthcare providers, yet substantial evidence for their educational efficacy for complex ordered procedures is still lacking. In the present study, the accuracy of pseudo-surgical instrumentation identification, modeled after perioperative nursing procedures, was compared following training through tablet-based simulations, text-based study, and real-world practice. The current results suggest notable benefits from tablet-based simulations, including enhanced object-oriented responding compared to text-based study, and increased practice repetitions compared to real-world practice over equivalent training time. Furthermore, this work demonstrated that knowledge acquired from tablet-simulations can be transferred to real-world tasks both immediately and at a delay, but that it may not replicate the full interactive benefits of practicing with real-world items immediately prior to the tested procedure. Overall, this study highlights the potential of simulated training methods in healthcare education, but suggests that caution should be used in their implementation.

## Supporting information

S1 Table Pseudo-surgical instrumentation naming systems. (DOCX)
