# Supplementary material for: Assessing the efficacy of tablet-based simulations for learning pseudo-surgical instrumentation
Source: PLoS One. 2021 Jan 14;16(1):e0245330. doi: 10.1371/journal.pone.0245330 (PMC7808648; doi:10.1371/journal.pone.0245330)
Supplement: S1 Table — (DOCX) [file pone.0245330.s001.docx]

***S1 Table:*** *Pseudo-surgical instrumentation naming systems*

| **Instrument Set** | **Item Family** | **Exemplars** |
| --- | --- | --- |
|  |  |  |
| **Set 1** | Crux | *Deca, Micro* |
|  | Edgit | *Deca, Micro* |
|  | Kalix | *M, F* |
|  | Azul __hold | *Micro, Macro* |
|  | Argentum | *Kosch, Hoyne, Doan, Wheat* |
|  | Mark | *Glonts, Garbin, Tress, Lugo, Tura* |
|  | Padding | *Malone, Raya, Ruth, Pauline, Olga, Mary* |
|  | Blouw __tines | *Sera, Duo, Tetra, Nila* |
|  | Embosser | *Solder, Bambose, Parasol, Amoretti, Juice* |
|  | Lignor | *Rute, Lignor* |
|  | Tray | *Quintal* |
|  |  |  |
|  |  |  |
| **Set 2** | Astera | *Centi* |
|  | Imbus | *Male, Female* |
|  | Lenar | *Centi, Nano* |
|  | Imbudo | *Da, Chone, Sheow* |
|  | Auric | *Lungit, Peekit, Grosset, Madiet, Mariet* |
|  | Excator | *Verdes, Azul, Rosa* |
|  | Fellstrip | *Avian, Pillar, Arachnian, Roberts, Gord, Boa* |
|  | Grune __dendors | *Poly, Quadro, Bi, Mono* |
|  | Pick | *Chronos* |
|  | Abraider | *Rene* |
|  | Reflector | *Grimhilde* |
|  | Press | *Ball, Demicore, Ravice, Amygdalar, Gully-basin* |
|  |  |  |
|  |  |  |
| **Set 3** | Lumix | *Milli* |
|  | Hexar | *M, F* |
|  | Slot | *Milli, Pico* |
|  | Bar | *Viga* |
|  | Clamp | *Pila, Verde, Sheow Blanche, Chone Blanche* |
|  | Fleratome | *Cas, Chus, Lans* |
|  | Microspear | *Visha* |
|  | Ribbon | *Fell, Hakin* |
|  | Fastener | *Bedsim, Hummel* |
|  | Hanimen | *Round, Square* |
|  | Scoop | *Truff* |
|  | __hesive __gauze | *Circum micro, Circum macro, Trans micro, Circum macro* |
|  | Svats __pode | *Rho, Delta, Beta, Alpha* |
|  | Printer | *Alai-B, Alai-G, Femoral-B, Femoral-G, Univalve-B, Univalve-G* |
|  |  |  |
